# Effect of membrane rigidification on the *BrAFP1* expression and cold-tolerance in *Brassica rapa*

**DOI:** 10.3389/fpls.2025.1527754

**Published:** 2025-08-13

**Authors:** Xiaoyun Dong, Jinxiong Wang, Jiaping Wei, Guoqiang Zheng, Zefeng Wu, Junmei Cui, Xuezhen Yang, Baojin Li, Shujun Zhu, Ermei Sa, Fengpeng Yang, Zigang Liu

**Affiliations:** ^1^ State Key Laboratory of Aridland Crop Science, College of Agronomy, Gansu Agricultural University, Lanzhou, China; ^2^ Agricultural Research Institute, Tibet Academy of Agriculture and Animal Husbandry Sciences, Lasa, China; ^3^ Seed Industry Development Section, Pingliang Seed Station, Pingliang, China; ^4^ Research and Development Center, Gansu Seed Industry Company Limited, Lanzhou, China

**Keywords:** membrane fluidity, cold tolerance, dimethyl sulfoxide, benzyl alcohol, proBrAFP1 activity

## Abstract

**Introduction:**

The cold tolerance of winter rapeseed cultivars is critically important for winter survival and yield formation in northern area. BrAFP1, an antifreeze protein in *Brassica rapa*, is hypothesized to stabilize membranes and inhibit ice crystal formation.

**Methods:**

we cloned the *BrAFP1* promoter from the cold-tolerant cultivar Longyou 7 (L7) and constructed the *proBrAFP1::GUS* expression vector to investigate the impact of membrane state changes on BrAFP1 expression and the cold tolerance in winter rapeseed. Ten independent transgenic T3 lines were generated, among which T3-5 and T3-7 were selected for subsequent analysis.

**Results:**

The dimethyl sulfoxide (DMSO) treatment in the absence of cold exposure activated the transcriptional activity of proBrAFP1, a cold-inducible promoter; in contrast, benzyl alcohol (BA) treatment eliminated its cold-induced activation. The expression levels of cold-responsive genes, including cyclic nucleotide-gated channel 1 (*CNGC1*), open stomata 1 (*OST1*), and inducer of CBF expression 1 (*ICE1*), as well as membrane fluidity-related genes, such as acyl-lipid desaturase 2 (*ADS2*), fatty acid desaturase 2 (*FAD2*), and sensitive to freezing 2 (*SFR2*), were significantly increased following DMSO pretreatment, while BA treatment significantly inhibited the expression of these genes. Furthermore, ABA and SA levels are closely linked to alterations in the membrane state, compared to untreated plants, the levels of ABA and SA in the leaves markedly increased at 4°C after DMSO and BA treatment but decreased at -4°C.

**Conclusion:**

Collectively, DMSO pretreatment enhanced cold tolerance, while BA pretreatment improved cell survival under cold stress, which is important for practise of keeping the rapeseed yields.

## 1 Introduction

Rapeseed is the second largest oilseed crop globally and a primary source of vegetable oil in China (Yang et al., 2019). In northern China, approximately 3.33 million hm² of winter fallow fields are available for cultivating winter rapeseed, which could yield an additional 10 million tons of rapeseed. This fact presents significant potential for both winter rapeseed cultivation and addressing the shortage of edible vegetable oil in China (Li et al., 2023). However, overwintering crop species or varieties must possess strong cold tolerance to survive the harsh winter conditions, particularly in the frigid winters of north-western China (Yan et al., 2006).

Freezing stress during overwintering leads to the formation of ice crystals within cells, dehydration of the protoplast, and the deterioration of the plasma membrane (Li et al., 2022). Overwintering crops grown in temperate regions have evolved various mechanisms to adapt to low temperatures, enabling them to survive under freezing conditions. Cold signaling involves a combination of chemical and physical cues that traverse the membrane, highlighting the plasma membrane’s critical role in perceiving cold signals from the environment and in enhancing cold tolerance in overwintering crops (Guo et al., 2018). The plasma membrane stability has been used as measure of temperature-stress tolerance in plants. The membrane fluidity is one of the fundamental characteristics derived from the functional components of membrane and the interactions among components,which determines the stability of the membrane (Tang et al., 2016). Both the long-term stability and short-term dynamics of the plasma membrane are essential for adapting to various stresses. Fluctuations in ambient temperature can alter membrane fluidity, with cold temperatures often affecting the membrane’s status and reducing fluidity. However, the precise relationship between changes in plasma membrane status, the expression of genes involved in cold signaling, and cold tolerance remains unclear (Tian et al., 2022).

When plants are exposed to temperatures below 0°C, the plasma membrane undergoes a combined physical and chemical attack (Guo et al., 2018). Low temperatures lead to a decrease in membrane fluidity and a loss of membrane integrity. The reduction in membrane fluidity results from physical cues, including decreased kinetic energy and the restricted movement of lipid molecules within the bilayer (Tan et al., 2023). Membrane integrity is compromised primarily due to chemical cues, such as reactive oxygen species (ROS), including superoxide radicals (O_2_^-^), hydrogen peroxide (H_2_O_2_), and hydroxyl radicals (·OH), which cause bursts of oxidative stress and damage to phospholipids (Xie et al., 2017; Sun et al., 2023). These physical and chemical signals induce changes in the membrane’s state under freezing conditions, thereby allowing cold signals to be transduced into cells and activating the expression of cold-responsive genes in the downstream cold signaling pathway. Membrane fluidity is considered a key factor in plant cold tolerance (Zhu et al., 2022).

FAD2 is a lipid desaturase, which can reduce the degree of lipids to maintain membrane fluidity under freezing condition (Zhou et al., 2022). The CNGC1 localized at the plasma membrane is one of a large family of non-selective cation-conducting channels controlling Ca^2+^ influx across membrane (Zhao et al., 2018; Guo et al., 2018). Cold is perceived by the rigidify state of membrane that then trigger the Ca^2+^ influx to activate downstream components, such as OST1, inducer of ICE1, CBF and COR. ICE1-CBF-COR pathway is a key regulator in cold-responsive pathway (Liu et al., 2018). OST1 phosphorylates ICE1 to activate its transcriptional activity, and then phosphorylated ICE1 can bind to the promoter of CBFs and initiate the CBFs transcription. A set of cold-regulated genes (*COR*), including antifreeze proteins, ROS scavengers, proteins related to regulation of osmotic substance synthesis and membrane stability, were activated by binding of CBF with promoters of *COR*s. Antifreeze protein (AFP) is one of CORs, which can specifically adsorb to the surface of ice crystals to inhibit recrystallization and growth of ice crystals (Dong et al., 2020). Therefore, AFPs can protect the cell membrane integrity and biomacromolecular structure from physical damages caused by large ice crystals in extracellular space (Eickhoff et al., 2019; Liu et al., 2019).

Winter rapeseed grown in temperate zones is exposed to freezing temperatures during overwintering (Liu et al., 2022). While changes in the plasma membrane state are recognized as an important factor in plant cold signal perception and cold acclimation (Sun et al., 2014; Todde et al., 2015; Kar et al., 2016), it remains unclear whether fluctuations in plasma membrane fluidity influence the cold tolerance of winter rapeseed (Dong et al., 2023). To investigate the effect of changes in membrane fluidity on cold tolerance, the transgenic *proBrAFP1::GUS Arabidopsis* seedlings and winter rapeseed were pretreated with either the membrane “rigidifier” DMSO or the membrane “fluidizer” BA. The pretreatment aimed to modulate membrane fluidity and assess its influence on cold tolerance mechanisms. The expression of *BrAFP1* was activated, and cold tolerance in seedlings was enhanced by DMSO at normal temperature. In contrast, both the expression of *BrAFP1* and cold tolerance were reduced by BA at low temperature.

## 2 Materials and methods

### 2.1 Plant material, growth conditions, and stress treatments

#### 2.1.1 Winter oilseed rape growing conditions

Seeds of two winter rapeseed cultivars, Longyou 7 (L7), which is highly resistant to cold damage with 99% overwinter survival below -10°C, and Tianyou 4 (T4), which is cold-sensitive with 52% overwinter survival below -10°C, were provided by the Key Laboratory of Crop Genetics Improvement and Germplasm Enhancement of Gansu Province, Lanzhou. Uniform, healthy seeds with excellent germination were selected and sown in pots filled with a 3:1 mixture of nutrient soil and vermiculite. The seedlings were grown under normal conditions in a light incubator until reaching the 5–6 leaf stage (incubation conditions: 20 ± 2°C, 16/8-h photoperiod, and 350 μmol photon m^-2^s^-1^).

At the 5–6 leaf stage, seedlings were subjected to foliar spraying treatments with the membrane rigidifier DMSO with 4%, the membrane fluidizer BA with 7 mM, or purified water (control). The plants were kept at normal temperature for 10 h after treatment. The treated plants were then divided into four groups and placed in a cold incubator for cold treatment at 4°C, -4°C, and -8°C for 24 h, with a 20°C treatment serving as the control (CK). Following treatment, various parameters were assessed, including relative electrolytic leakage (REL), soluble protein content, hormone levels, malondialdehyde (MDA), superoxide anion (O_2_^-^), hydrogen peroxide (H_2_O_2_), antioxidant enzyme activity, and the expression levels of BrAFP1 and cold-related genes, which were measured by qRT-PCR.

#### 2.1.2 Cultivation Conditions for Arabidopsis

Sterilized seeds of T3–5 and T3–7 were placed on filter paper saturated with sterile water and incubated in a growth chamber under the following conditions: 20°C, light intensity of 180 μmol photon m^-2^s^-1^, relative humidity of 70-80%, and a 16/8-h photoperiod. After 7 days, the seedlings were transferred to Petri dishes containing filter paper soaked with chemical solutions (the chemicals used were DMSO at concentrations of 0%, 2%, and 4%, and benzyl alcohol (BA) at concentrations of 3.5 mM, 7 mM, and 14 mM) and treated for 10 h. After treatment, 7-day-old seedlings were gently transferred using forceps to filter paper soaked with sterile water and incubated at 4°C for 24 h for GUS staining.

Sterilized seeds were sown evenly using sterile toothpicks into moist soil (nutrient soil: vermiculite = 3:1) and incubated for 21 days under the following conditions: temperature 20°C, light intensity of 180 μmol photon m^-2^s^-1^, relative humidity of 70-80%, and a 16/8-h photoperiod. After 21 days, seedlings were treated with foliar spraying of DMSO (4%), benzyl alcohol (BA, 7 mM), or purified water (control). These treatments were identical to those applied to the winter oilseed rape. Following treatment, the plants were divided into two groups and placed at 4°C for 24 h. The 21-day-old treated seedlings were then harvested in three biological replicates for qRT-PCR assays or GUS staining.

### 2.2 Construction of promoter reporter plasmids and transformation of *Arabidopsis*

The specific primers of pSP-F/pSP-R were designed against the 1332 bp sequence of the *BrAFP1* promoter (forward primer: 5′- ccatgatctacagcgctgaagcttTTAACACATCCACTTATTAGCCTTA-3′, and reverse primer: 5′- gactgaccacccggggatccATGCTACATAAGCTTAAGAG-3′), in which the *Bam*HI and *Hind*III restriction sites have been introduced and are underlined here. L7 leaf genomic DNA was used as a template for polymerase chain reaction (PCR) amplification to obtain the target fragment. The TWV1-GUS vector is double digested with *Hind*III and *Bam*HI and recycled before recombining with the target fragment.

The *proBrAFP*::GUS positive plasmid was transferred into *Agrobacterium* strain GV3101 by the freeze-thaw method and cultured in liquid LB (Holsters et al., 1978), the bacterial precipitate was collected and resuspended in 5% sucrose solution (current allotment) with 0.01% of the surfactant silwet L-77 added. The prepared *Agrobacterium* solution was introduced into *Arabidopsis* by the floral dip method (Clough and Bent, 1998). Seeds from transformed T0 plants were harvested, sterilised with 3% sodium hypochlorite and sown on MS culture medium supplemented with hygromycin (20 mg/mL). Plates were placed in the dark at 4°C for 2–3 days. Plates were then transferred to a long-day light cycle (16 h light/8 h dark) at 20 ± 2°C. Seven-day-old positive plants, screened by resistance seedlings, were transferred to soil and grown to the T1 generation. T1 genomic DNA was extracted from leaves via the CTAB method and PCR detection was performed using HPT-specific primers hpt-F (5′-ACACTACATGGCGTGATTTCAT-3′) and hpt-R (5′- TCCACTATCGGCGAGTACTTCT-3′). T2 seeds were obtained in the same way as T1 seeds. Homozygous proBrAFP1 lines (T3–5 and T3-7) in the T3 generation were further identified by hygromycin resistance screening and PCR.

### 2.3 Total RNA extraction and quantitative RT-PCR analysis

The RNA was extracted from leaf samples of three biological replicas using the Steady Pure Plant RNA extraction kit (Accurate Biotechnology, Changsha, China, AG21019-S) following the manufacturer’s protocol with some modification. Subsequently, use M-MLV Reverse Transcriptase Reverse Transcription Kit (AG11706) to obtain single-chain cDNA for RT-PCR (Accurate Biotechnology, Changsha, China, AG11706). The cDNA concentration was determined using an ultra-micro UV spectrophotometer (Nanopro 2010/2020) and then stored at -20°C.

SYBR^®^ Green Premix *Pro* Taq HS qPCR (Rox Plus) Kit (AG11718, Accurate Biology, China) was used for the qRT-PCR analysis, and the total reaction system consisted of 2 μL of cDNA template, 0.8 μL each of upstream and downstream primers, 10.0 μL of SYBR^®^ Green Pro Tax, and ddH_2_O to a total volume of 20 μL. The qRT-PCR conditions were specified initial denaturation at 95°C for 30 seconds, 40 cycles of denaturation at 95°C for 5 seconds and annealing at 60°C for 30 seconds. The dissolution curve analysis was kept as default, and 95°C was continued for 1 second. The comparative cycle threshold method was used to analyze the data. The relative gene expression values were calculated using the comparative cycle threshold (Ct) method (Livak and Schmittgen, 2001). Three biological replicates with four technical replicates were carried out for each treatment. Tubulin and Actin were used as internal references for *Arabidopsis thaliana* and *Brassica rapa*, respectively. All of the specific primers for qRT-PCR designed using the software Primer Premier 5, and synthesized by Xian Qingke Biotechnology Company and the sequences are shown in **Supplementary Table 1**.

### 2.4 Determination of plant endogenous hormone content

A slightly modified high performance liquid chromatography (HPLC) was used for the determination of plant hormones. The 0.1 g leaf samples were ground to fine powder in liquid nitrogen with a pestle and mortar, and 6 mL of pre-chilled extraction solution (n-propanol: H_2_O: HCl = 2:1:0.002) was added and the mixture was placed in an ice bath at 100 rpm for 30 min. Next, 2 mL of chromatography-grade methylene chloride was added, and the solution was vortexed for 10 s, shaken in ice bath at 100 rpm for 30 min and then centrifuged at 13000 rpm for 6 min at 4°C. The supernatant was transferred to a new 15 mL centrifuge tube and then concentrated and dried at room temperature under nitrogen, before it was re-dissolved by adding 400 µL of the prepared 80% methanol solution and vortexed for 10 s. Centrifuge the samples in a microfuge at 13000 rpm for 15 minutes at 4°C. Finally, the supernatant was carefully removed using a syringe with a needle, filtered through a 0.22 um membrane filter and injected directly into an injection vial for analysis.

The chromatographic conditions were as follows: Agilent ZORBAX SB-C18 (5 mum, 4.6 mm x 250 mm); detection wavelength at 254 nm; mobile phase: 100% methanol and 0.1% aqueous phosphoric acid; flow rate, 1.0 mL/min; column temperature, 30°C; and detection temperature was room temperature and injection was auto injection with a volume of 10 µL.

### 2.5 Measurement of physiological and biochemical indicators

Relative electrolytic leakage determination experiments were measured using the method described by (Zhang et al., 2018), with a slight modification was used to measure relative electric conductivity. Measurements of soluble protein content (SP), superoxide dismutase (SOD), peroxidase (POD), catalase (CAT), and ascorbate peroxidase (APX) activity were performed using the UV spectrophotometer method (Zhang et al., 2006). The SP content was determined using the Coomassie brilliant blue G-250 staining method (Bradford, 1976), the SOD activity was measured by the nitroblue tetrazolium (NBT) method, and the POD activity was measured by the guaiacol method (Dong et al., 2019). The MDA content was measured by the thiobarbituric acid (TBA) method (Song et al., 2011), the H_2_O_2_ and O^2-^ content of samples were measured using kits from Suzhou Comin Biotechnology Co. (H_2_O_2_ -1-Y kit and SA-1-G kit).

### 2.6 Histochemical staining

Histochemical staining of superoxide anion (O^2-^) and hydrogen peroxide (H_2_O_2_) was done using nitro blue tetrazolium chloride (NBT) and 3,3-diaminobenzidine (DAB) according to the method of Daudi and O’Brien (2012) with slight improvements. GUS histochemical staining was performed using a GUS staining kit (Beijing, Coolaber Technology Co., SL7160).

The trypan blue staining method was modified slightly based on the method by Yamaguchi et al. (2012). The leaf discs were punched into 0.5 cm diameter discs and placed in 15 mL centrifuge tubes, and 5 mL of trypan blue mixture (60 mL ethanol, 10 mg trypan blue, 10 mL phenol, 10 mL glycerol, 10 mL lactic acid, 10 mL distilled water) was added and boiled for 2–3 min after 10 min of resting, cooled at room temperature, then rinsed with sterile water, and decoloured with 2.5 g/ml of chloral hydrate for 1–2 days, and then observed under a stereomicroscope, before being stored in 60% glycerol at 4°C.

### 2.7 Data analysis and statistics

Physiological data were analyzed with SPSS 19.0 software using one-way analysis of variance with least significant difference test and Duncan’s multiple range test at 0.05 level. All results are presented as means and standard errors of the mean. Graphs were generated using Origin 8.0, and figures were assembled using Adobe Illustrator software (Adobe, CA, USA).

## 3 Results

### 3.1 Generation of *proBrAFP1::GUS* transgenic lines of *Arabidopsis*

**Figure 1:**
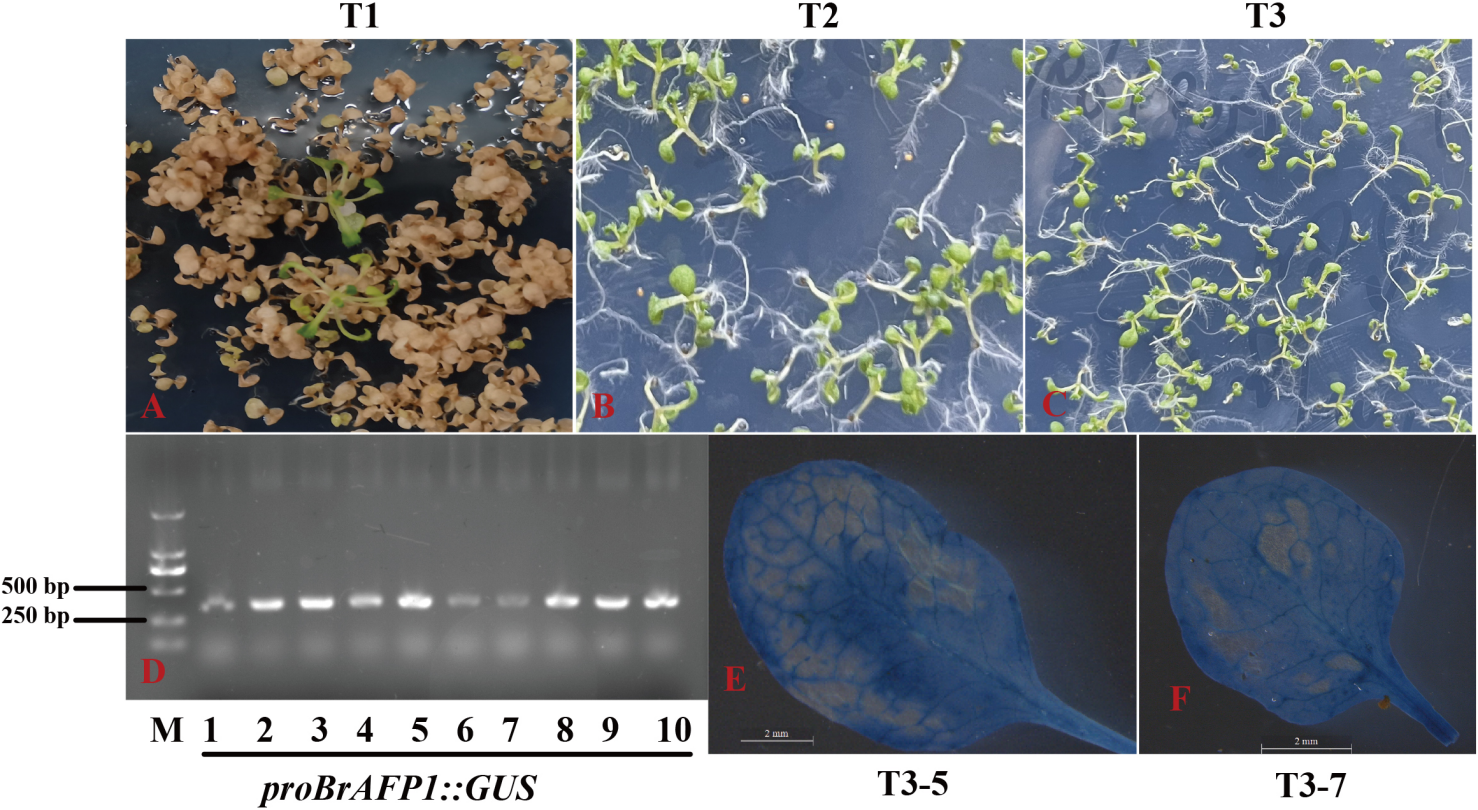
Identifying of *proBrAFP1::GUS* transgenic positive *Arabidopsis* plants. Screening of transgenic positive lines using hygromycin resistance (Hyg resistance) in T1 **(A)**, T2 **(B)**, and T3 **(C)** generations. **(D)** PCR amplification for the GUS gene fragment in transgenic lines. GUS staining in leaves of two T3 transgenic lines, T3-5 **(E)** and T3-7 **(F)**.

To investigate the impact of plasma membrane fluidity changes on the expression of *BrAFP1* gene in the cold-tolerance of winter rapeseed (*Brassica rapa*), we cloned the promoter of *BrAFP1* from Longyou 7 (L7) that is a strong cold-tolerant cultivar of winter rapeseed. The promoter was then linked to the TWV1-GUS vector, constructing a recombinant plasmid of *proBrAFP1::GUS*, which was introduced into the GV3101 strain of *Agrobacterium tumefaciens* and then transformed into *Arabidopsis thaliana*. For the selection of transgenic lines of *Arabidopsis*, Hyg-resistance screening (**Figures 1A–C**) and GUS staining assays were performed. Subsequently, we obtained ten transgenic T3 lines (T3–1 to T3-10) from independent transformation events. Among these, lines T3–5 and T3–7 exhibited deeper GUS staining in leaves after cold treatment (**Figures 1E, F**) and were thus selected for further experiments.

### 3.2 Membrane rigidification induces *proBrAFP1* activity and enhances cold tolerance of plants

**Figure 2:**
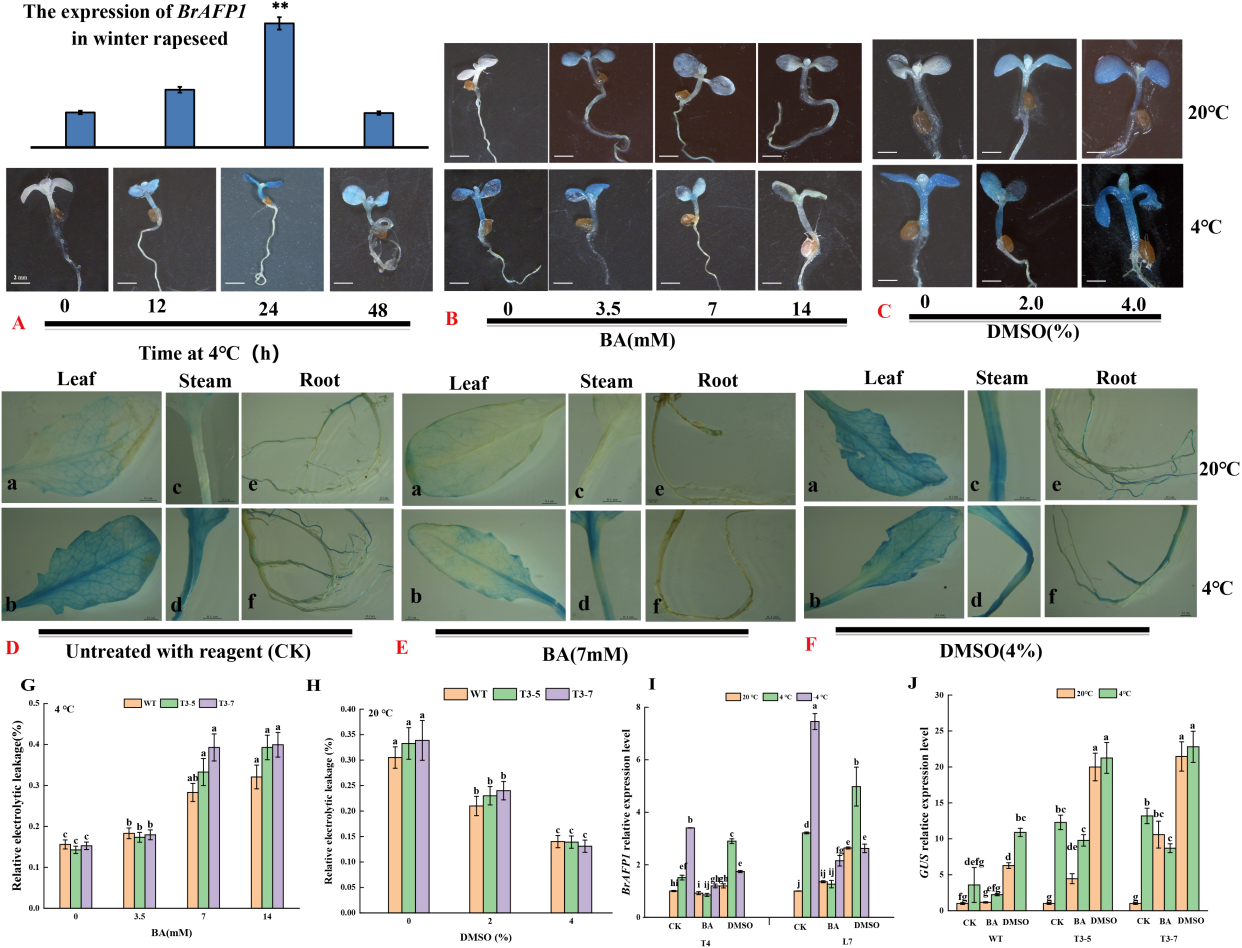
Histochemical GUS assay of *pBrAFP1::GUS* transgenic *Arabidopsis* seedlings. **(A)** The transcript level of *AFP1* in transgenic *Arabidopsis* seedlings at low temperature of 0,12, 24, and 48 h; **(B)** The transcript level of *AFP1* in transgenic *Arabidopsis* seedlings treated with 0, 3.5, 7, and 14 mM BA; **(C)** The transcript level of *AFP1* in transgenic *Arabidopsis* seedlings treated with 0, 2.0, and 4.0% DMSO; The transcript level of *AFP1* in different tissues (leaf, steam, root) of transgenic *Arabidopsis* treated with ddH_2_O **(D)**, 7 mM BA **(E)** and 4.0% DMSO **(F)**, respectively; The relative electrolytic leakage of transgenic *Arabidopsis* treated with BA **(G)** and DMSO **(H)**; **(I)** The expression levels of Br*AFP1* in T4 and L7 treated with ddH_2_O, 7 mM BA and 4.0% DMSO; **(J)** The expression levels of *GUS* gene in transgenic *Arabidopsis* treated with ddH_2_O, 7 mM BA and 4.0% DMSO. The values are means ± standard deviation from four biological replicates (p<0.05).

To determine the optimal cold duration for BA and DMSO pretreatment, transgenic *Arabidopsis thaliana* seedlings were placed at 4°C for different duration (0 to 48 h). Subsequently, the seedlings were transferred into trypan blue solution for GUS staining analysis. The results of the GUS staining showed that 24 h cold-treated seedlings had the deepest color, indicating effective proBrAFP1 activity induction (**Figure 2A**). This cold-treatment duration was selected for subsequent experiments.

To examine whether membrane rigidification affects proBrAFP1 activity as an indicator of cold signaling, transgenic lines were treated with DMSO or BA as a membrane rigidifier and fluidizer, respectively, then those samples were transferred into trypan blue solution for GUS staining analysis. The stems and leaves of transgenic lines were stained dark blue in the cold-treatment (**Figure 2A**). Similar results were also observed in both 2% and 4% DMSO pretreatments without cold-treatment (**Figures 2C, J**). In contrast, seedlings of transgenic lines treated with the fluidizer BA at low temperatures showed light staining, and with the staining intensity decreasing as the BA concentration increased (**Figures 2B, J**). These results obtained from studying mature plants of the transgenic lines were consistent with those observed in the seedlings (**Figures 2D–F**).

When winter-type Chinese cabbage (*Brassica rapa*) was treated with DMSO or BA, the transcription level of *BrAFP1* in the leaves of young plants significantly increased in DMSO treatment at normal temperature (**Figure 2I**). In contrast, *BrAFP1* transcription levels markedly decreased in the leaves treated with BA at low temperature (**Figure 2I**). These findings indicate that membrane rigidification is essential for the induction of *proBrAFP1* activity.

The relative electrolytic leakage, as a key indicator for assessing cold tolerance, reflects plasma membrane integrity suffering abiotic stresses. When the seedlings pretreated with either DMSO or BA and subsequently exposed to low-temperature at 4 °C for 24h, the REL of transgenic lines and WT significantly decreased in the 2% and 4% DMSO pretreatment compared to the non-pretreated seedlings (**Figure 2H**). In contrast, the electrolytic leakage of seedlings pretreated with BA significantly increased compared to the contrast without BA pretreatment (**Figure 2G**). This result indicates that DMSO pretreatment can improve plant cold tolerance.

### 3.3 Effect of membrane reagent treatment on the expression of genes in the cold-signal pathway

The SFR2, ADS2, and FAD2 are key enzymes in membrane fatty acid metabolism, essential for maintaining membrane structural stability. The CNGC is one of plasma membrane channel for Ca^2+^ as a second messenger, playing an important role in cold signal transduction. OST1 phosphorylates ICE1 to activate its transcriptional activity. The ICE1-CBF pathway is at the core of the cold signal regulatory network. CBF further regulates the cold-responsive gene *COR*, of which *BrAFP1* is a notable member.

**Figure 3:**
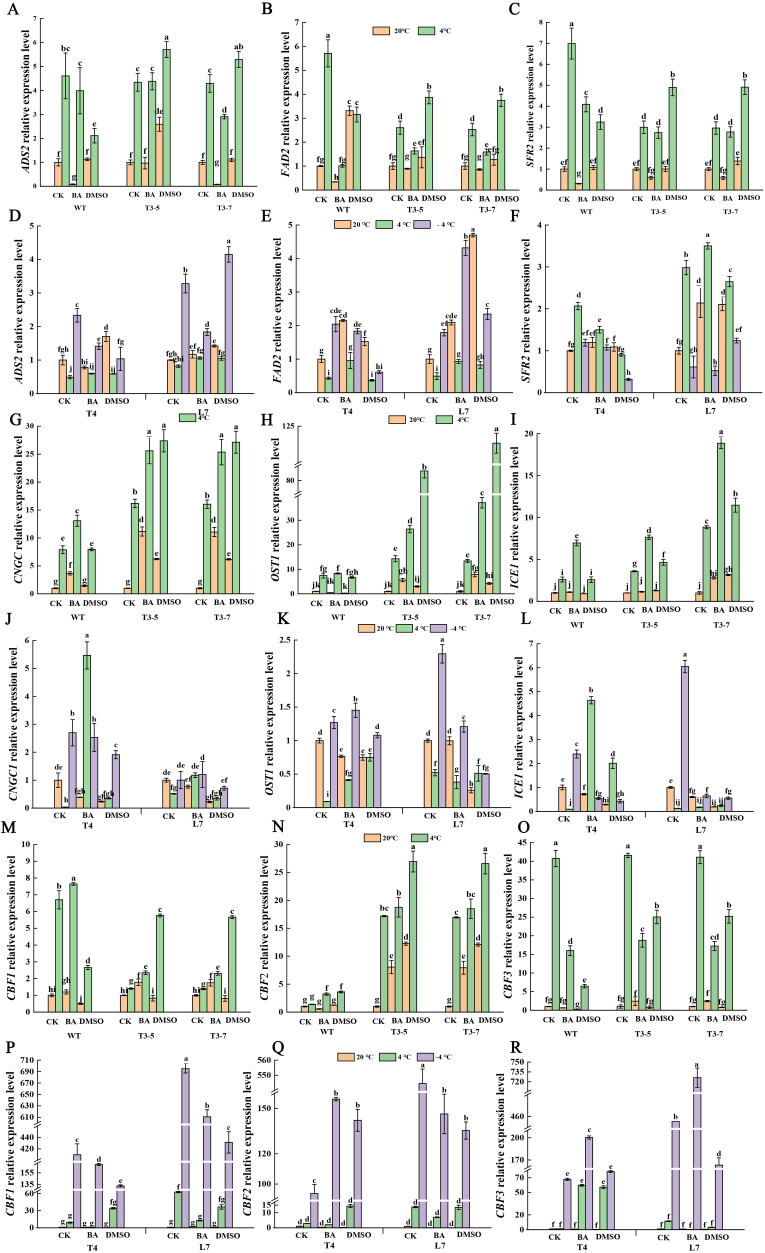
Effect of membrane state intervention reagents on the transcription levels of key genes in cold signaling pathway. The expression levels of *ADS2* **(A, D)**, *FAD2* **(B, E)**, *SFR2* **(C, F)**, *CNGC* **(G, J)**, *OST1* **(H, K)**, *ICE1* **(I, L)**, *CBF1* **(M, P)**, *CBF2* **(N, Q)**, *CBF3* **(O, R)** in transgenic Arabidopsis thaliana and in winter rapeseed, respectively. The values are means ± standard deviation from four biological replicates (p<0.05). Different lowercase letters.

The transcription levels of the *ADS2*, *FAD2* and *SFR2* genes in *Arabidopsis thaliana* were elevated in pretreatment with DMSO without cold-treatment (**Figures 3A–C**); notably, the increase in FAD2 reached a statistically significant level (**Figure 3B**). In contrast, pretreatment with BA markedly inhibited the expression of these three genes in *Arabidopsis* at normal temperature (**Figures 3A–C**). Following DMSO pretreatment, cold-treatment further enhanced the aforementioned trend in gene expression in plants of transgenic lines, meaning that the increase in gene expression levels in transgenic plants pretreated was greater than in plants that were not pretreated at low temperature (**Figures 3A–C**). However, in wild-type plants that pretreated with DMSO, the expression levels of these three genes at low temperature were lower than in those without pretreatment, which is puzzling. In *Brassica rapa* winter rapeseed, the expression of these three genes showed a similar pattern (**Figures 3D–F**) to that observed in transgenic lines of *Arabidopsis* preteated with DMSO without cold-treatment.

Under ambient temperature conditions, pretreatment with DMSO and BA significantly upregulated *CNGC1* gene expression in *Arabidopsis thaliana*. Following low-temperature exposure, seedlings that had been pretreated exhibited an even greater increase in *CNGC1* expression compared to untreated plants (**Figure 3G**). In *Brassica*, however, the response differed. Pretreatment with DMSO and BA led to a clear downregulation of *CNGC1* expression at both ambient temperature and -4°C. At 4°C, BA-pretreated seedlings showed a notable increase in *CNGC1* expression, while DMSO-treated L7 seedlings displayed a slight decrease (**Figure 3J**). After DMSO and BA pretreatment, low-temperature conditions led to a significant increase in the expression of *OST1* and *ICE1* genes in both T3 lines compared to those without pretreatment, excluding the WT line (**Figures 3K, L**). Under ambient temperature, those genes in the T3 lines showed either a slight or significant increase in expression (**Figures 3H, I**).

In *Arabidopsis*, after DMSO and BA pretreatment, low-temperature exposure led to an increased expression of *CBF1* and *CBF2* genes compared to plants without pretreatment, except in the WT treated with DMSO (**Figures 3M, N**). The increase was more pronounced in seedlings treated with DMSO than those treated with BA (**Figures 3M–O**). At ambient temperature, DMSO pretreatment resulted in a slight or significant decrease in *CBF1* expression in seedlings, whereas BA pretreatment caused a slight or significant increase in *CBF1* expression (**Figure 3M**). For *CBF2*, both pretreatments significantly increased expression, with the increase being greater in DMSO-treated seedlings (**Figure 3N**). Following pretreatment with both agents, *CBF3* expression was significantly reduced under low-temperature conditions, while its expression remained relatively unchanged at ambient temperature (**Figure 3O**).

In winter rapeseed, pretreatment with DMSO and BA led to a significant decrease in *CBF1* expression at -4°C (**Figure 3P**). In BA-pretreated T4 and L7 seedlings, *CBF3* expression significantly increased, whereas in DMSO-pretreated seedlings, *CBF3* expression slightly increased in T4 but significantly decreased in L7 (**Figure 3R**). For *CBF2*, pretreatment with both agents lead to a significant increase in expression in T4 seedlings, while expression in L7 seedlings was significantly reduced compared to untreated plants (**Figure 3Q**). At ambient temperature, the treatment with both agents had no significant effect on *CBFs* gene expression (**Figures 3P–R**).

### 3.4 Effect of membrane reagent pretreatment on endogenous hormone content in leaves of winter rapeseed

**Figure 4:**
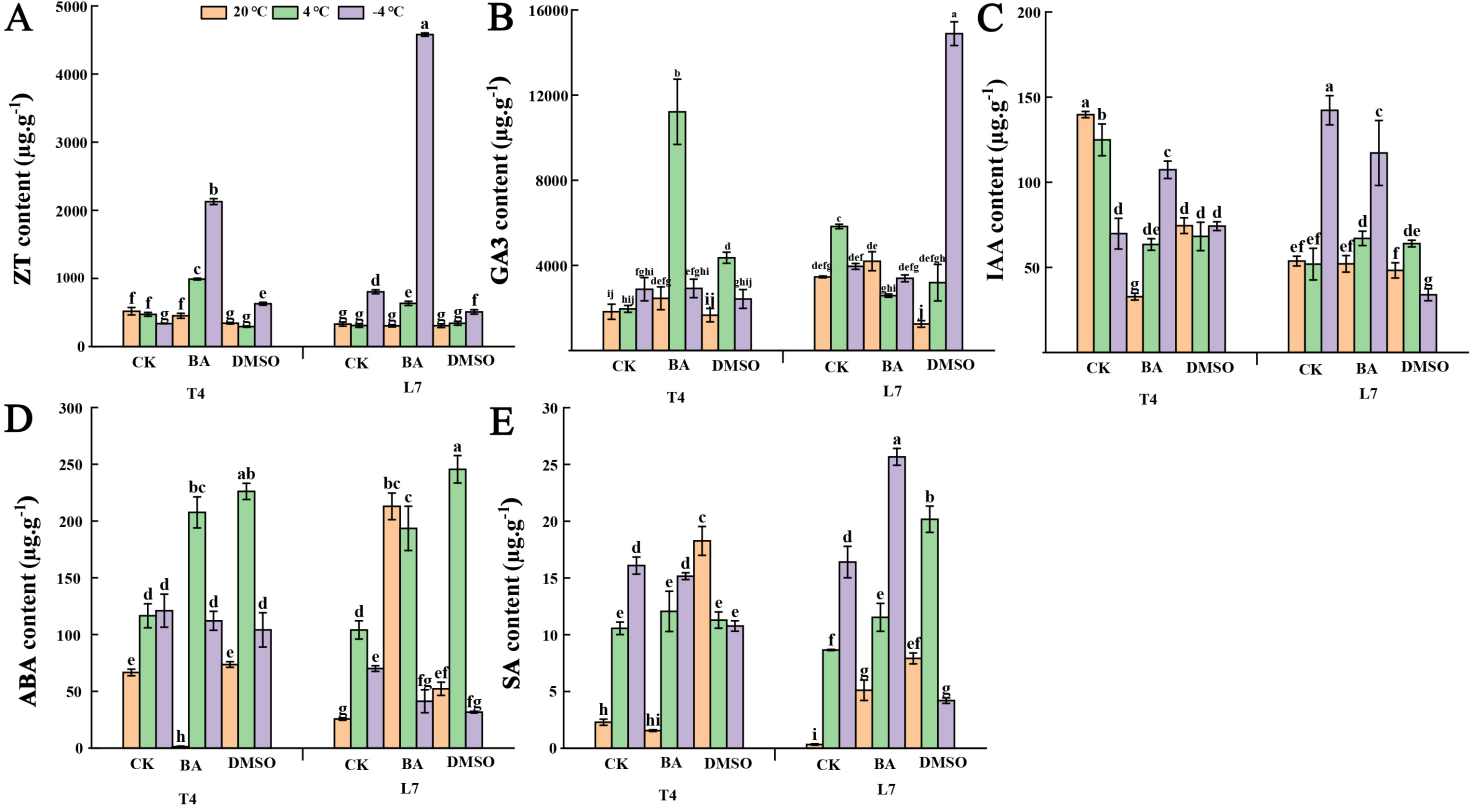
Effect of membrane state intervention reagents on the content of endogenous hormone in winter rapeseed. **(A)** zeatin (ZT); **(B)** gibberellin (GA3); **(C)** auxin (IAA); **(D)** abscisic acid (ABA); **(E)** salicylic acid (SA). The values are means ± standard deviation from four biological replicates (p<0.05). Different lowercase letters.

After BA pretreatment, the ZT content in the leaves of winter rapeseed significantly increased under low-temperature conditions (**Figure 4A**). In T4 leaves, GA3 content showed a slight increase at -4°C and a significant increase at 4°C (**Figure 4B**). In contrast, DMSO-pretreated leaves exhibited slight or significant reductions in ZT, GA3, and IAA content, except for ZT in T4 leaves and GA3 in L7 leaves at -4°C. (**Figures 4A–C**) These results suggest that membrane solidification (via DMSO pretreatment) inhibits growth hormone accumulation in winter rapeseed, while increased membrane fluidity (via BA pretreatment) promotes it. Additionally, both DMSO and BA pretreatments led to a significant increase in ABA and SA content in leaves at 4°C compared to untreated controls. However, at -4°C, ABA and SA levels generally decreased slightly or significantly, with the exception of a significant increase in L7 leaves pretreated with BA. Under normal temperatures, both DMSO and BA pretreatments significantly increased ABA and SA in L7 leaves, while BA pretreatment reduced these hormone levels in T4 leaves. DMSO pretreatment resulted in higher hormone levels at ambient temperatures compared to untreated leaves (**Figures 4D, E**). These findings indicate that ABA and SA levels in L7 leaves are more responsive to changes in membrane state, suggesting an enhanced ability for adaptive adjustment in response to membrane alterations.

### 3.5 Effect of membrane reagent treatment on the oxidative state of winter rapeseed

**Figure 5:**
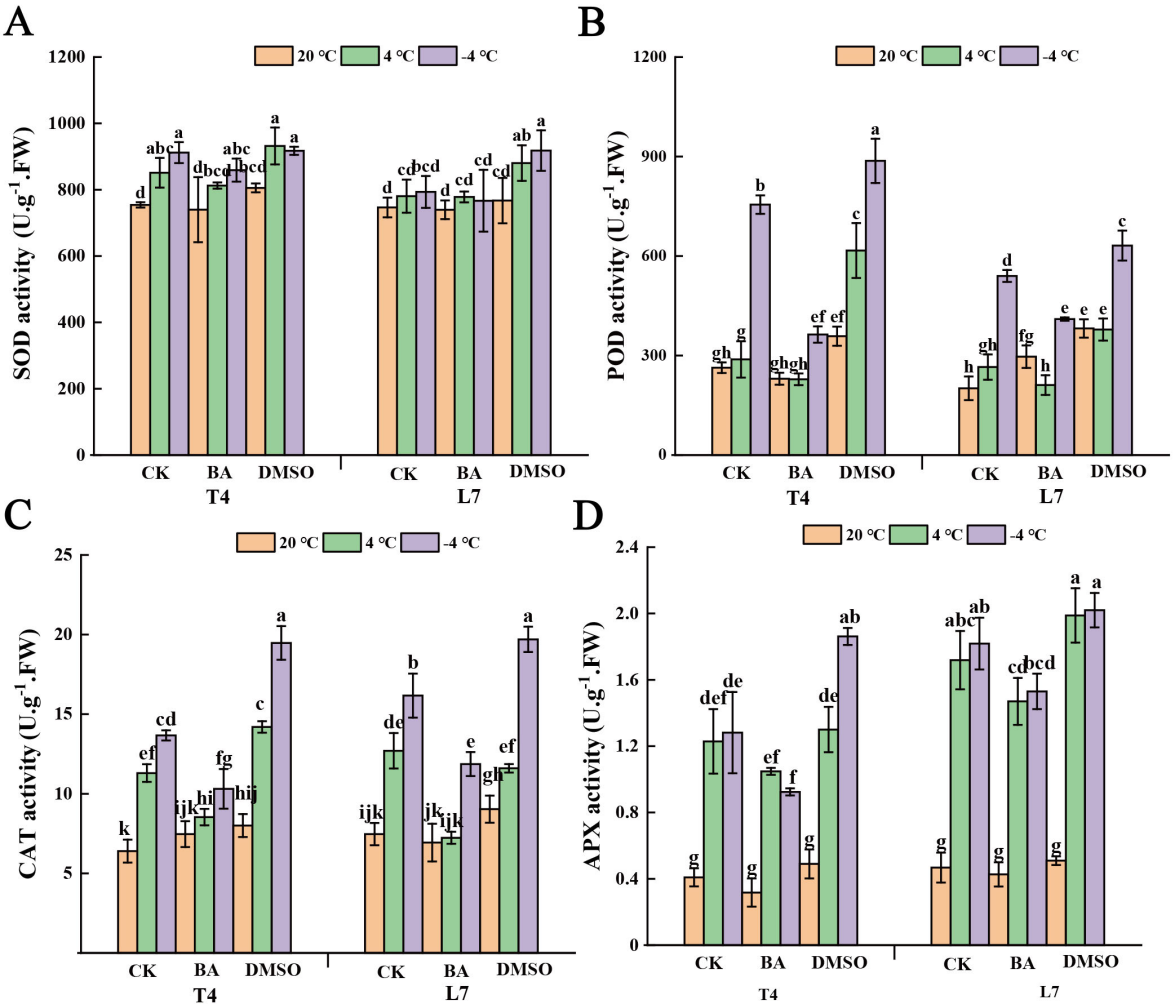
Effect of membrane state intervention reagents on antioxidant enzymes activities of winter rapeseed. The activities of **(A)** superoxide dismutase (SOD), **(B)** peroxidase (POD), **(C)** catalase (CAT) and **(D)** ascorbate peroxidase (APX) in the leaves of L7 and T4 under different temperatures by membrane reagents BA and DMSO. The values are means ± standard deviation from four biological replicates (p<0.05). Different lowercase letters.

After DMSO pretreatment, the SOD activity in L7 leaves was significantly higher at both 4°C and -4°C compared to BA-pretreated and untreated leaves, while BA pretreatment showed no notable effect on SOD activity (**Figure 5A**). At ambient temperature, neither pretreatment had a significant effect on SOD activity in the leaves (**Figure 5A**). Additionally, POD, CAT and APX activities in winter rapeseed leaves increased significantly following DMSO pretreatment. Under low-temperature conditions, BA-pretreated leaves exhibited notably higher POD, CAT and APX activities than untreated leaves (**Figure 5B–D**).

**Figure 6:**
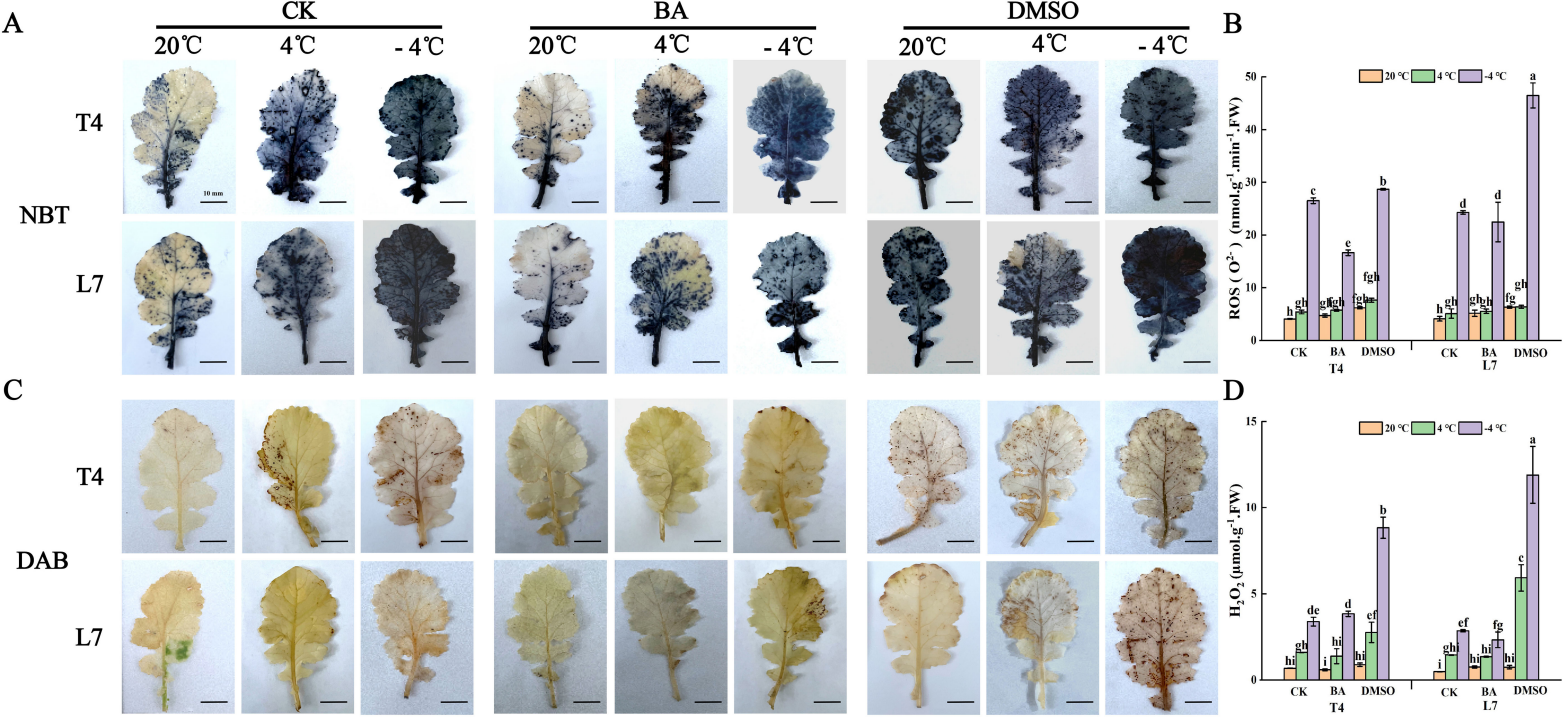
Effect of membrane state intervention reagents on the oxidative state and cell survival of winter rapeseed leaves. A, C are NBT **(A)** and DAB **(C)** staining of winter rapeseed leaves, respectively. Scale bar, 1 cm. The same below. O^2-^ **(B)**, and H_2_O_2_ **(D)** content accumulation in leaves of L7 and T4 under different temperatures by membrane reagents BA and DMSO. Scale bars=10mm; The values are means ± standard deviation from four biological replicates (p<0.05). Different lowercase letters.

After NBT (nitroblue tetrazolium chloride) staining, blue spots on the leaves indicate the distribution and accumulation of superoxide anions (O_2_^-^); while DAB staining reveals hydrogen peroxide accumulation as dark brown spots (Wu et al., 2015). In winter rapeseed, DMSO pretreatment led to substantial O_2_^-^ accumulation in leaves at ambient temperature, with even higher levels observed under low-temperature treatment. In contrast, BA pretreatment significantly reduced O_2_^-^ content in the leaves compared to untreated plants (**Figure 6A**). Under low-temperature conditions, DMSO pretreatment led to a higher accumulation of hydrogen peroxide in leaves of winter rapeseed compared to untreated plants, while BA pretreatment showed no significant change in hydrogen peroxide levels. At ambient temperature, neither DMSO nor BA pretreatment had a noticeable effect on hydrogen peroxide content in the leaves (**Figure 6C**). These findings were further confirmed by quantitative measurements of the superoxide anion content and hydrogen peroxide levels in the leaves (**Figures 6B, D**).

At ambient temperature, DMSO pretreatment significantly increased CAT activity in winter rapeseed leaves, with an even greater increase observed at -4°C compared to untreated plants. In contrast, BA pretreatment led to a slight increase in CAT activity at ambient temperature, but a significant decrease under low-temperature conditions. Under low-temperature conditions, APX activity in DMSO-pretreated leaves increased markedly, whereas BA pretreatment caused a notable reduction in APX activity. At ambient temperature, pretreatment with either DMSO or BA had little effect on APX activity in the leaves.

### 3.6 Effect of membrane reagent treatment on osmotic regulation substances of winter rapeseed

**Figure 7:**
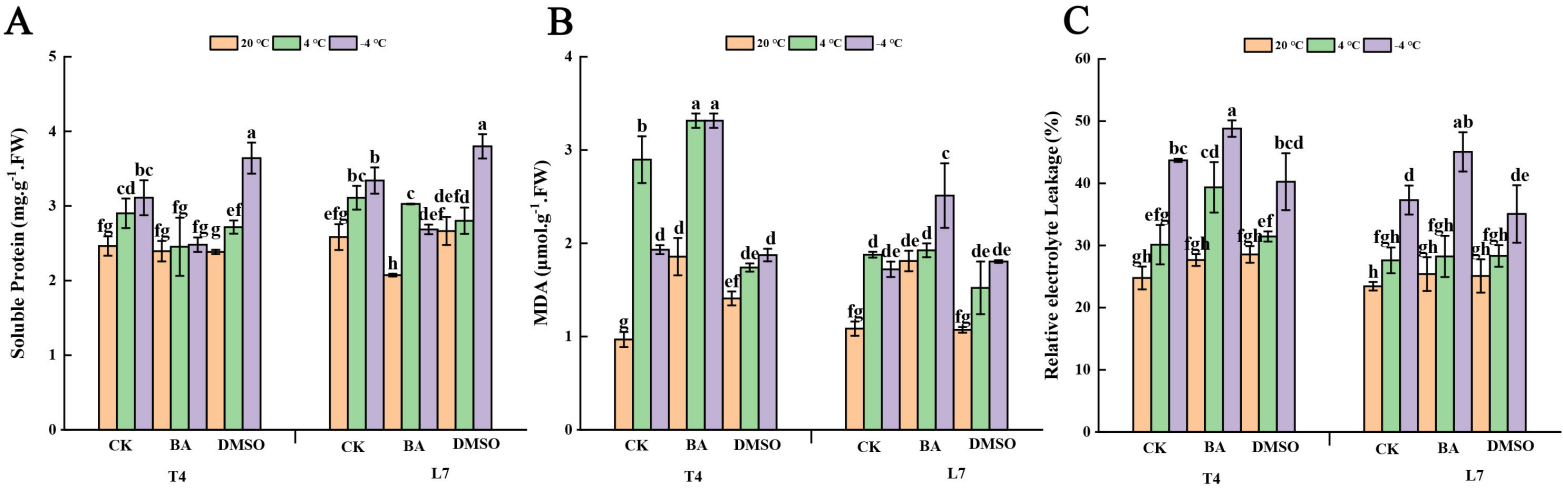
Effect of membrane intervention reagent on the content of osmotic adjustment substances in winter rapeseed. The changes of soluble protein (**A**, SP), malondialdehyde (**B**, MDA) and relative electrolytic leakage (**C**, REL) in leaves of L7 and T4 under different temperatures by membrane reagents BA and DMSO. The values are means ± standard deviation from three biological replicates (p<0.05). Different lowercase letters.

After DMSO pretreatment, compared with the control, the content of soluble protein, which serves as an osmoregulatory substance in the leaves of winter rapeseed, significantly increased at -4°C and notably decreased at 4°C. The pretreatment at ambient temperature had little effect on changes in soluble protein content (**Figure 7A**). Compared with the control, BA pretreatment led to a significant decrease in soluble protein content under low-temperature conditions (**Figure 7A**). After DMSO pretreatment, the MDA content of T4 leaves exhibited significant changes compared to the control under both 4°C and ambient temperature. In contrast, the relative electrolyte leakage and MDA content in L7 leaves remained largely stable (**Figures 7B, C**). However, BA pretreatment led to a slight or significant increase in relative electrolyte leakage and MDA content relative to the control (**Figures 7B, C**). This might be one of the main reasons why L7 is cold-resistant. These results suggest that DMSO pretreatment may play a role in alleviating osmotic stress induced by subzero temperatures.

### 3.7 Effect of membrane reagent pretreatment on cell viability of winter rapeseed leaves

**Figure 8:**
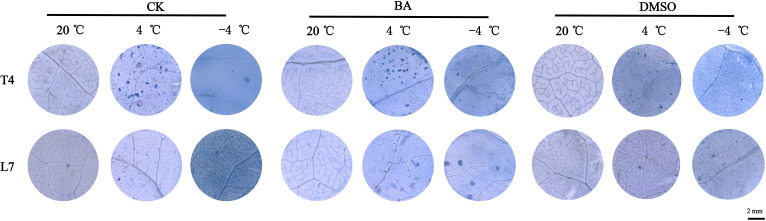
Phenotype diagram of trypan blue staining in rapeseed leaves. Scale bar=2 mm.

The trypan blue staining is a common method for assessing cell viability, where dead cell clumps appear as blue patches (Yamaguchi et al., 2012). After DMSO application, the leaves of winter rapeseed showed deeper staining and more blue patches, while BA application resulted in lighter staining and fewer patches compared to untreated leaves (**Figure 8**). This finding indicates that membrane solidification at low temperatures has a negative impact on cell survival, while fluidization pretreatment can improve cellular cold tolerance.

## 4 Discussion

### 4.1 Membrane rigidification activates *BrAFP1* expression in winter rapeseed

In northern China, winter rapeseed faces the challenge of harsh winter cold, with its stems and leaves directly exposed to the atmosphere (Dong et al., 2020), making these parts highly sensitive to environmental changes (Liu et al., 2019). Under freezing conditions, BrAFP1, an antifreeze protein induced in the aerial stems and leaves of winter rapeseed, specifically binds with high affinity to the surfaces of ice crystals (Dong et al., 2023). Cold-induced synthesis of BrAFP1 exhibits strong recrystallization inhibition activity, effectively preventing the formation of large ice crystals within the tissues and thereby protecting the cell membrane systems and biomolecules from mechanical damage caused by large ice crystals (Liu et al., 2019). When temperatures drop, plants sense cold signals through changes in the fluidity of the phospholipid bilayer in cell membranes, activating membrane proteins such as CNGCs, which mediate Ca^2+^ influx into intracellular space from the extracellular Ca^2+^ reservoir (Guo et al., 2018). The intracellular cold signal is transmitted via the OST1-ICE1-CBF pathway, which is central to the cold signaling network (Liu et al., 2018), activating downstream COR (cold-responsive) genes and conferring cold acclimation traits to the plant. *BrAFP1*, a member of the *COR* gene family in *Brassica rapa*, is a cold-inducible gene. In this study, seedlings of winter rapeseed and *proBrAFP1::GUS* transgenic *Arabidopsis* lines were treated with the membrane rigidifier DMSO and the fluidizer BA. DMSO pretreatment without cold-treatment significantly increased transcriptional activity of *CNGC*, *OST1*, *ICE1*, and *CBF2* genes in seedlings of both winter rapeseed and transgenic lines. Furthermore, the *BrAFP1* expression was negatively correlated with REL after BA and DMSO pretreatments (**Table 1**). Additionally, *proBrAFP1* was cold-activated in stems and leaves of transgenic seedlings, with a marked increase in *BrAFP1* expression in winter rapeseed seedlings. These findings suggest that membrane rigidification can activate the cold signaling pathway in winter rapeseed, indicating that it is an essential component of the cold signaling network in *Brassica rapa*.

**Table 1:** Correlation analysis between *BrAFP1* and cold resistance indexes.

	REL	H2O2	O2-
BrAFP1	-.945**	-0.212	-0.385

### 4.2 Membrane rigidification accelerates the cold acclimation of winter rapeseed

In optimal environmental conditions, various reactive oxygen species (ROS) are generated as byproducts of metabolic processes within plant cells (Liu et al., 2021). To maintain cellular homeostasis, intracellular antioxidant enzyme systems, including oxidases and other protective enzymes, neutralize ROS, sustaining a dynamic balance in ROS metabolism (Zheng et al., 2022). However, under mild environmental stresses, this ROS balance is disrupted, leading to increased ROS production and intracellular accumulation (Zheng et al., 2023). ROS accumulation serves as a cellular signal for stress, initiating multiple downstream responses such as osmolyte synthesis and accumulation for osmotic regulation, and activation of oxidase systems for adjustments to maintain oxidative metabolism equilibrium (Liu et al., 2025). Conversely, when stress intensity exceeds the plant’s tolerance threshold, severe metabolic imbalances trigger ROS bursts, ultimately causing cellular damage (Guo et al., 2018). In this study, DMSO pretreatment, a membrane rigidifier, without low-temperature treatment led to significant ROS accumulation in winter rapeseed leaves, specifically in O^2-^ and H_2_O_2_ levels, accompanied by a marked increase in CAT activity. Under low temperatures, ROS accumulation was pronounced, and the activities of POD, CAT, and APX were significantly enhanced. In contrast, pretreatment with BA followed by low temperature led to a noticeable reduction in ROS accumulation. With low temperatures causing a natural decrease in plasma membrane fluidity and a tendency for membrane solidification, DMSO pretreatment further intensified the stress intensity, simulating an increase in the severity of cold stress. This resulted in a rapid rise in ROS accumulation in winter rapeseed leaves. Although the elevated ROS levels caused some cellular damage, ROS accumulation also promoted osmolyte synthesis and enhanced the activity of protective enzymes such as POD, which helped mitigate lipid peroxidation and preserve plasma membrane integrity. Consequently, the cold acclimation process in winter rapeseed was accelerated, suggesting that membrane rigidification under stress conditions may play a key role in inducing ROS-mediated responses that strengthen cellular resilience against cold-induced damage.

## 5 Conclusion

This study demonstrates that DMSO-induced membrane rigidification enhances the expression of the antifreeze protein BrAFP1, upregulates cold-responsive genes, and improves osmotic regulation and antioxidant enzymes activity under low-temperature conditions, thereby promoting cold tolerance. Conversely, BA-mediated membrane fluidization results in diminished *BrAFP1* expression, impaired cold signal transduction, and reduced cell viability under cold stress. These observations underscore the importance of maintaining an optimal membrane state for effective cold acclimation and survival. Additionally, our findings suggest that modulating membrane fluidity represents a promising strategy for enhancing cold tolerance in crops. Future investigations should aim to elucidate the molecular mechanisms governing membrane state regulation and explore their potential applications in agricultural biotechnology.

## Data Availability

The original contributions presented in the study are included in the article/**Supplementary Material**. Further inquiries can be directed to the corresponding author.
