# Supplementary material for: Effect of membrane rigidification on the BrAFP1 expression and cold-tolerance in Brassica rapa
Source: Front Plant Sci. 2025 Aug 13;16:1527754. doi: 10.3389/fpls.2025.1527754 (PMC12380914; doi:10.3389/fpls.2025.1527754)
Supplement: Supplementary file 1 [file Table1.docx]

Supplementary table 1 The list of all primers used in this study

| Primer name | Sequence（5′-3′） |
| --- | --- |
| At-GUS-F | CGACAGCAGCAGTTTCATCAATCAC |
| At-GUS-F | ACTCCTACCGTACCTCGCATTACC |
| At-ADS2-F | CTTGGTCGGCTCTTTGGGTTACG |
| At-ADS2-R | GCGAGAAGGGCACAATAGGCTAAG |
| At-AFP-F | GCCACTGACACCACCACTGATAC |
| At-AFP-R | AGGGTAGAGATTCACGAGCAAGGG |
| At-CBF1-F | CGGCTTGGCGGCTACGAATC |
| At-CBF1-R | ACCATCGTCTCCTCCATGTCCAG |
| At-CBF2-F | GACCTTGGTGGAGGCTATTT |
| At-CBF2-R | ATCCCTTCGGCCATGTTATC |
| At-CBF3-F | TCAATTTCGCTGACTCGGCTTGG |
| At-CBF3-R | CCGTCGTCGCATCACACATCTC |
| At-CDPK-F | GTCTGGAGTGCTGGAGTGATTGTG |
| At-CDPK-R | TCACCGTGGAGGACCTGTTCG |
| At-CNGC-1-F | GAAACCAAGGGAACCGAAGA |
| At-CNGC-1-R | GCCATGGAACACGCTTTAAC |
| At-FAD2-F | TCTCTCCTCCCTCAGCCTCTCTC |
| At-FAD2-R | ACCAACTGTGTCATCCAGCCATTG |
| At-ICE1-F | GTCAACTCCTCCTGGATCTTTG |
| At-ICE1-R | GAGGGACACAACTCTTCCTTG |
| At-OST1-F | ACTGTTGGAACTCCTGCTTAC |
| At-OST1-R | CTCGGGATCTTCGAAAGGATATG |
| At-SFR2-F | CACTGGATGGCTGTTGCTCACTC |
| At-SFR2-R | GGTCGCATAAAGGAGACGTGGTG |
| At-Actin-F | GTGTCATGGTTGGGATGGGT |
| At-Actin-R | AAGAACCGGGTGCTCTTCAG |
| BrAFP1-F | TGGGAGCCTTCACGGACACG |
| BrAFP1-R | GCAGAGGAGATCGCTTGTTCACC |
| Br-ADS2-F | GCTGGTCGGCTCTTTGGGTTAC |
| Br-ADS2-R | GCGAGAAGGGCACAATAGGCTAAG |
| Br-CBF1-F | CTGCGGGTCGGAAGAAGTTTCG |
| Br-CBF1-R | CTTGTTTGGCTCCCTCACCTCAC |
| Br-CBF2-F | GACGATCGTGGAGGCTATTT |
| Br-CBF2-R | GCATTCCTTCCGCCATACTA |
| Br-CBF3-F | GGCAACGGCAGTGGCTTCTC |
| Br-CBF3-R | ACAGCCTCCACCATTGTCTCCTC |
| Br-CDPK-F | CTGATGTGTGGAGTGCTGGAGTG |
| Br-CDPK-R | GTCAAGATCACCGTGGAGAACCTG |
| Br-CNGC-1-F | ACAACGGTCGCTATCTGAATAC |
| Br-CNGC-1-R | TCTACGATCCCTGACCTCAAT |
| Br-FAD2-F | GCGTTGGCTACCGTTGACAGAG |
| Br-FAD2-R | GCGTGGTCGAGAACAGGTGATG |
| Br-ICE1-F | GTTCAGAACGGAGGAGGTAAAG |
| Br-ICE1-R | GGGACAACTGATCTAAGCATGTA |
| Br-OST1-F | ACATCGAGAGAGGTGAGAAGA |
| Br-OST1-R | CTCCAGCTGCGTATTCCATAA |
| Br-SFR2-F | CGGACTTCTTGGCACGATCACC |
| Br-SFR2-R | TGAAATCGGCGAGGACTTCTTTGG |
| Hyg(417)-F | AAATCCGCGTGCACGAGGT |
| Hyg(417)-R | TCGTTATGTTTATCGGCACTTTGCA |
